# An Entropy-Based Approach to Portfolio Optimization

**DOI:** 10.3390/e22030332

**Published:** 2020-03-14

**Authors:** Peter Joseph Mercurio, Yuehua Wu, Hong Xie

**Affiliations:** 1 Department of Mathematics and Statistics, York University, Toronto, ON M3J 1P3, Canada; 2 Manulife Financial Corp, Toronto, ON M4W 1E5, Canada

**Keywords:** return entropy, portfolio optimization, entropy portfolio optimization, portfolio selection, Markowitz mean variance, investment risk, modern portfolio theory, capital asset pricing model, diversification

## Abstract

This paper presents an improved method of applying entropy as a risk in portfolio optimization. A new family of portfolio optimization problems called the return-entropy portfolio optimization (REPO) is introduced that simplifies the computation of portfolio entropy using a combinatorial approach. REPO addresses five main practical concerns with the mean-variance portfolio optimization (MVPO). Pioneered by Harry Markowitz, MVPO revolutionized the financial industry as the first formal mathematical approach to risk-averse investing. REPO uses a mean-entropy objective function instead of the mean-variance objective function used in MVPO. REPO also simplifies the portfolio entropy calculation by utilizing combinatorial generating functions in the optimization objective function. REPO and MVPO were compared by emulating competing portfolios over historical data and REPO significantly outperformed MVPO in a strong majority of cases.

## 1. Introduction

Markowitz [1] introduced the world’s first fundamentally sound quantitative approach to portfolio selection in 1952. He proposed an algorithm that finds the optimal capital allocation across a set of assets based on user-controlled risk parameters. Investors were suddenly given the mathematical tools needed to construct data-driven optimal portfolios according to their preferred risk tolerances. Based on the volatility of random returns, Markowitz’s mean-variance portfolio optimization (MVPO) measures the risk of an asset by its second central moment: the variance or squared deviation of returns from the mean. In the multiple-asset case, the risk of a portfolio is measured by the covariance of returns of its comprised assets weighted by their respective capital allocations. The result is a personally-tailored investment portfolio with the optimal balance between risk and return. Further work on the topic was contributed by Tobin (1958) [2]. Over the years that followed, MVPO quickly became the de facto standard for portfolio selection and capital asset pricing among institutional equity firms, mutual funds, and hedge funds. Its massive influence led to the term *variance* becoming ubiquitous when evaluating risk in the world of finance. Markowitz’s variance-based approach to risk mitigation formed the foundation for modern portfolio theory and investment analysis, and inspired the basis for the capital asset pricing model (CAPM) introduced independently by Sharpe (1964) [3], Lintner (1965) [4,5], and Mossin (1966) [6].

More recently, topical literature has explored some common difficulties encountered when employing MVPO in the real world. Notably, there are five main issues that complicate the use of MVPO in practice: (i) optimal solutions assigning large allocation weights to high risk assets, (ii) disturbance of the assets’ dependence structure, (iii) drastic variations in optimal solutions when adjusting inputs, (iv) accommodation of non-normal or asymmetric returns, and (v) difficulty in estimating the covariance matrix and expected returns. Researchers have suggested various solutions for addressing these main issues. One popular method is the Black–Litterman asset allocation model (1991, 1992) [7,8] which allows investors to input their own “investor views” without causing unexpected results. A particularly promising approach was to use entropy as a risk instead of variance, first proposed by McGill (1954) [9] and Garner (1956) [10], and then extended to the portfolio selection problem by Philippatos (1972) [11]. Philippatos’ use of joint entropy resulted in a complex computation that proved to be a road-block for practical applications. A main focus of contemporary literature on this topic explored entropy of the portfolio weights as a maximization objective to encourage diversification levels, as found in work by Cheng (2006) [12], Usta (2007) [13] Huang (2007, 2008, 2012) [14,15,16], and Palo (2016) [17]. A Kullback–Leibler view of maximum entropy is demonstrated by Abbas (2017) [18], and the use of cross-entropy minimization was explored by Post (2017) [19]. Further details on the use of entropy for portfolio selection are discussed in Section 2. Nevertheless, we believe that entropy-based risk is an ideal approach to addressing the five main difficulties with MVPO. The key is developing a simple yet effective method for calculating the entropy of a portfolio. More detailed discussion is presented in Section 3. The paper is thus organized as follows: Section 2 provides a brief review of relevant portfolio optimization methods to date. Section 3 introduces the concept of entropy as a risk measure and its favorability as an approach to portfolio optimization, and then details the featured method of this paper: return-entropy portfolio optimization (REPO). A real-life portfolio selection example using REPO is demonstrated in Section 4, and conclusions are discussed in Section 5.

Throughout this paper, if the size of the distribution support of a discrete random variable is *m*, we say that the distribution or the random variable has *m* states.

## 2. Modern Portfolio Theory

### 2.1. Markowitz Mean-Variance Portfolio Optimization (MVPO)

The portfolio selection problem can be stated as such: given a set of *n* assets and their respective expected future returns $E\left(R_{1}\right),\ldots ,E\left(R_{n}\right)$, the goal is to construct the optimal portfolio $R_{P}$ by allocating weights $w_{1},\ldots ,w_{n}$ representing the percentages of capital to invest into each asset. The objective function of this optimization problem is designed to minimize the risk and maximize the expected returns of the portfolio. Markowitz (1952) [1] defined risk as the variance of the portfolio returns. Markowitz’s MVPO minimizes variance and maximizes expected returns via the following multi-objective function and constraint set:

(1) $$\begin{array}{cc} \mathrm{minimize}\;\; & \mathrm{Var}\left(R_{P}\right)=w_{1}^{2}\sigma_{1}^{2}+\cdots +w_{n}^{2}\sigma_{n}^{2}+\sum_{i}\sum_{j\neq i}w_{i}w_{j}\sigma_{i}\sigma_{j}\rho_{ij}\\ \mathrm{maximize}\;\; & E\left(R_{P}\right)=w_{1}E\left(R_{1}\right)+\cdots +w_{n}E\left(R_{n}\right)\\ \mathrm{subject}\;\;\mathrm{to}\;\; & w_{1}+\cdots +w_{n}=1,\;\;\;w_{i}\geq 0\;\;\forall \;i, \end{array}$$

where $\sigma_{k}^{2}$ is the variance of $R_{k}$, and $R_{P}=w_{1}R_{1}+\cdots +w_{n}R_{n}$.

### 2.2. Practical Difficulties with MVPO

There are five main practical difficulties that are often encountered when utilizing MVPO in the real world. These are:

(1) Large weights assigned to high risk assets (sparse solution). In practice, the mean-variance optimization tends to concentrate large-percentage allocations on few assets, often ones with high risk. This is especially common when adjusting the risk parameter to achieve greater returns. This creates a sparse solution with little diversification, which is a consequence opposed to the original intention. This challenge has been studied by various authors who tried to improve the mean-variance portfolio diversification. See Black (1992) [8], Green (1992) [20], Corvalán (2005) [21], and Koumou (2019) [22]. Shannon entropy became a popular method in the sense of diversifying the portfolio weights, and further details on this are found in Section 2.3. Diversification using different entropy measures were explored by Yu (2014) [23]. Lastly, approaches using Rao’s quadratic entropy and diversity measures (Rao, 1982, 1985, 2004, 2010) [24,25,26,27,28] are discussed in detail by Carmichael (2015) [29].

(2) Disturbing the dependence structure equilibrium. An investor using MVPO typically calculates a covariance matrix of historical returns for the risk function, but may often wish to input his/her own views (estimates/opinions) about future expected returns. Using investor views instead of historical returns can disturb the dependence structure equilibrium and cause unexpected optimization results, such as (3). See Black (1992) [8] and Babaei (2015) [30].

(3) Drastic variations in optimal solutions when adjusting inputs. An important consequence of (2) is that there are drastic variations in optimal solutions when taking investors’ views into consideration. Small changes in the expected return inputs can cause major changes in optimal solutions, which is counterintuitive and unpredictable. See Michaud (1989) [31], Best (1991) [32], Jorion (1992) [33], and Chopra (1993) [34].

(4) Dealing with returns that are non-normal or asymmetric. The Markowitz model relies on symmetry and normality assumptions, and departure from these assumptions can lead to unexpected results. See Jondeau (2005) [35] and Karandikar (2012) [36]. In the real world, asset returns are typically not normally distributed or even symmetric, which makes variance a poor measure for risk. This is not ideal for any investment strategies because an upside volatility is actually welcomed or even desired. Solutions to non-normality and asymmetry in the literature fall into two main categories:

1. Post-modern portfolio theory (PMPT): (i) It only minimizes the downside volatility; (ii) it considers asset distributions as log-normal instead of normal; (iii) it optimizes higher moments than variance (skewness and kurtosis). See Rom (1993) [37] and Sortino (1994) [38].

2. The portfolio entropy minimization method (entropy as a risk): (i) It does not require the normality assumption; (ii) it can accommodate asymmetric distributions; (iii) it is fully non-parametric. See Philippatos (1972) [11], Jiang (2018) [39], and Lassance (2019) [40]—further discussion in Section 2.3.

(5) Difficulty in estimating covariance matrix and expected returns. Since portfolio optimization is a forward-looking exercise, historical returns may not be very useful, as past returns are not always indicative of future returns. Forecasts for expected returns are often used instead (i.e., investors’ views of expected returns). The covariance matrix can also be difficult to estimate. See Wong (2012) [41] and Sun (2019) [42].

### 2.3. Literature Review

Various authors have managed to address one, two, or even three of these issues, but not all five at once. The following is a brief review of previous research on these topics.

Addressing (1) and (4), Philippatos (1972) [11] aimed to find the optimal portfolio by minimizing the portfolio entropy. Philippatos described three methods by which one can construct mean-entropy diversified portfolios: (i) by calculating the individual and conditional entropies and using them in conjunction with the expected returns; (ii) by computing the individual entropies for each security and their conditional entropies with respect to the level of some acceptable market index (diagonal-index model); and (iii) by computing the security and portfolio entropies directly from the respective variances when it can be assumed that the probability distribution of returns is known. For the first method, it is worth noting that a significant number of conditional entropies must be computed to obtain the joint entropy of the portfolio, since the joint entropy is comprised of the sum of the conditional entropies. For example, calculating the joint entropy of a four-security portfolio with only three states requires one individual entropy plus 3+9+27=39 conditional entropies. In general, $\sum_{i=0}^{n-1}m^{i}$ individual and conditional entropy calculations are required, where *n* is the number of securities and *m* is the number of probability states, as shown in the Appendix A. As a result of this complexity, the diagonal-index model was proposed by Philippatos. But this approach provides a poor approximation that does little to account for the true dependence structure of the assets. Lastly, the third method assumes some probability distributions, but is not applicable for unknown distributions or non-parametric approaches. Other measures of entropy, such as Rényi entropy (1960) [43], have been explored by Lassance (2019) [40], but involve similar reliance on a non-parametric estimator of the exponential Rényi entropy function.

Issues (2) and (3) gained the most attention from Black and Litterman at the Goldman Sachs Fixed Income team, who presented methods to help to preserve the dependence structure and stabilize solutions. The Black–Litterman model (Black, 1991, 1992) [7,8] allows users to provide their views that represent their opinions on expected returns and confidence levels. The post-modern portfolio theory was coined by software entrepreneurs Rom and Ferguson (1993) [37] with their work involving the downside risk that targets (4). Additionally, Sortino (1994) [38] introduced the Sortino ratio, which measures the downside-risk-adjusted returns of an asset or portfolio. Cheng (2006) [12], Usta (2007) [13], Huang [14,15,16], and Bera (2008) [44] all proposed maximum entropy diversification (MED) methods that maximize the entropy of the portfolio weight vector from MVPO, addressing (1). Palo (2016) [17] argues that portfolio risk and diversification should be managed distinctly, and empirically shows that entropy is a useful means to alleviate the lack of diversification of portfolios on the efficient frontier using a maximum entropy method like MED. A proposed solution called the risk parity given by Asness (2012) [45,46] was to extend the risk-free tangent line by borrowing capital and leveraging the portfolio. This is a simple and intuitive solution to (1) but does not address the other issues, and such leverage may not be feasible or available to all investors. Usta (2011) [47] extended the maximum entropy (diversification) approach with the mean-variance-skewness-entropy (MVSE) optimization by adding a multi-objective function to maximize portfolio skewness, which also targets (4). Fono (2011) [48] attempted to obtain an optimal portfolio by introducing semi-kurtosis into the objective function to minimize the low-side tail risk via the mean-semivariance-skewness-semikurtosis (MSSS) optimization. Urbanowicz (2014) [49] took the same approach to the diversification as Cheng and Huang but used Tsallis entropy of portfolio weights instead of Shannon entropy. These methods do not use the joint entropy, as they deal only with the one-dimensional entropy of the portfolio weights vector. A maximum entropy method was proposed by Xu (2014) [50] that aimed to maximize the worst-case portfolio returns. In recent years, entropy was used to evaluate tail risks by Geman (2015) [51]. Zhou (2015) [52] assumed independence between assets to approximate the portfolio entropy by the sum of the individual entropies. Zhou then accommodated the missing dependence structure by also minimizing the portfolio variance, via a multi-objective function. Most recently, Rotela (2017) [53] used the entropic data envelopment analysis (DEA) to improve the diversification of optimized portfolios. Zhou (2017) [54] evaluated six entropy-based risk measures and declared the mean fuzzy entropy optimization as the best performing method. Dai (2018) [55] used the concept of quadratic entropy to minimize the risk of a portfolio via the mean-quadratic entropy (MQE) optimization by using a multi-objective function that maximizes the entropy of the portfolio weights and minimizes the (approximate) quadratic entropy of the portfolio.

## 3. Entropy as a Risk Measure

### 3.1. Shannon Entropy (Information Theory)

In 1948, Shannon [56,57] introduced the concept of information entropy. Applied to a probability vector, the information entropy represents the amount of randomness or uncertainty inherent to that probability distribution: it is a measure of how many “choices” are involved in the selection of an event or of how certain we are to its outcome.

For a discrete random variable *X* with probability mass function P(·) that can take on possible values $x_{1},\ldots ,x_{n}$, the Shannon entropy *H* is the average amount of information produced by *X*, defined as:

(2) $$H\left(X\right)=E\left(-\log P\left(X\right)\right)=-\sum_{i=1}^{n}P\left(x_{i}\right)\log P\left(x_{i}\right).$$

For two discrete random variables *X* and *Y* respectively having *n* and *m* states, the joint entropy of *X* and *Y* is given by:

(3) $$H\left(X,Y\right)=-\sum_{i=1}^{n}\sum_{j=1}^{m}P_{XY}\left(x_{i},y_{j}\right)\log P_{XY}\left(x_{i},y_{j}\right).$$

Furthermore, the conditional entropy of *Y* given *X*, representing the average amount of information in *Y* given *X*, is defined by:

(4) $$H\left(Y|X\right)=-\sum_{i=1}^{n}\sum_{j=1}^{m}P_{XY}\left(x_{i},y_{j}\right)\log P_{Y|X}\left(y_{j}|x_{i}\right)=H\left(X,Y\right)-H\left(X\right).$$

While variance and entropy are both non-negative quantities, it is important to note a main differences between the two measures: variance takes a value in [0,∞), whereas the entropy of a random variable with *m* states is bounded by the “maximal entropy”; i.e., the entropy of the uniform distribution with *m* states, as shown by using Jensen’s inequality (Jensen, 1906) [58]:

(5) $$H\left(X\right)=E\left[\log\left(\frac{1}{P(X)}\right)\right]\leq \log\left(E\left[\frac{1}{P(X)}\right]\right)=\log\left(m\right).$$

### 3.2. Portfolio Optimization Based on Entropy

By using entropy in place of variance for the portfolio selection problem, all five main difficulties with MVPO can be solved, as (1) low risk portfolios selected by entropy-based methods provide greater diversification levels than those selected by variance-based methods; (2) the dependence structure is maintained, since the entropy is not based on the mean; (3) the optimal solution is more stable under the adjustments to investors’ views; (4) entropy is a non-parametric function designed to accommodate non-normality and asymmetry; and (5) no pre-calculations of any covariance matrices are necessary, as the joint entropy dependence structure can be automatically captured in the objective function.

The aim of this paper is to introduce a single optimization problem that solves all five issues with the MVPO method. Presented here is an approach to the portfolio optimization using a minimum entropy method, called return-entropy portfolio optimization (REPO), which has not yet been proposed elsewhere. As other methods encountered the difficulty in calculating the portfolio entropy due to the complexity of the joint entropy expression, REPO calculates the resulting portfolio entropy directly in the objective function using combinatorial generating functions, eliminating the need for any intermediary joint entropy calculations. It provides the following practical benefits over MVPO: better stability under changes to inputs, robustness against non-normality and asymmetry, and improved portfolio performance, as shown in Section 4.

### 3.3. Probability Generating Functions

Consider an asset’s return, a random variable *R*, and its historical observations $r=(r_{1},\ldots ,r_{T})$. The range of historical observations can be divided into *m* distinct probability state partitions, $A_{1},\ldots ,A_{m}$ with endpoints $\left[a_{0},a_{1}\right],\ldots ,\left[a_{m-1},a_{m}\right]$ respectively, such that each $r_{j}$ belongs to only one partition; i.e., $a_{k-1}<r_{j}\leq a_{k}$, for some integer *k* in 1,…,m. Without loss of generality we can assume the partitions to be equally sized. The probability of each event $\left\{R\in A_{k}\right\}$ can be estimated empirically over *T* time periods as:

(6) $$\hat{f_{T}}\left(r;k\right)=\frac{1}{T}\sum_{j=1}^{T}I\left(a_{k-1}<r_{j}\leq a_{k}\right)\approx P\left(R\in A_{k}\right).$$

Consider now a portfolio with two assets. Denote their returns by $R_{1},R_{2}$, respectively. Let their actual returns over *T* time units be $r_{1}=\left(r_{11},\ldots ,r_{1T}\right)$ and $r_{2}=\left(r_{21},\ldots ,r_{2T}\right)$, with portfolio weights $w_{1},w_{2}$ such that $w_{1}+w_{2}=1$. The portfolio entropy of $R_{P}=w_{1}R_{1}+w_{2}R_{2}$, $H(R_{P})$, can be estimated by using the empirical probability frequency tables directly via combinatorial generating functions as follows. Take the empirical probability generating function *g*:

(7) $$g\left(x;w_{1}r_{1}+w_{2}r_{2}\right)=\frac{1}{T}\sum_{j=1}^{T}x^{\left\{k\;:\;a_{k-1}<w_{1}r_{1j}+w_{2}r_{2j}\leq a_{k}\right\}},$$

for *k* such that $\left\{a_{k-1}<w_{1}r_{1j}+w_{2}r_{2j}\leq a_{k}\right\}$. Notice that to sum over all the powers of *x* one needs to count all the occurrences of the event $\left\{R_{P}\in A_{k}\right\}$ enumerated in the coefficients similarly to how a histogram or frequency table counts occurrences. The coefficient of $x^{k}$ estimates the empirical probability of event $\left\{R_{P}\in A_{k}\right\}$, $\hat{f_{T}}\left(r_{1},r_{2};k\right)$. These coefficients can be extracted for each polynomial term by taking the *k*th-derivative of *g* at x=0, $g^{\left(k\right)}\left(0\right)$, divided by k!. Now with the estimated probabilities of events, the empirical entropy can be calculated directly. Each coefficient of $x^{k}$ represents the estimated empirical probability of event $\left\{R_{P}\in A_{k}\right\}$, which is given by:

(8) $$\frac{g^{\left(k\right)}\left(0\right)}{k!}=\hat{f_{T}}\left(r_{1},r_{2};k\right)=\frac{1}{T}\sum_{j=1}^{T}I\left(a_{k-1}<w_{1}r_{1j}+w_{2}r_{2j}\leq a_{k}\right)\approx P\left(R_{P}\in A_{k}\right).$$

These estimated probabilities are then substituted into the formula for Shannon entropy (1948) [56,57] for *m* total probability states:

(9) $$H\left(R_{P}\right)=-\sum_{k=1}^{m}\hat{f_{T}}\left(r_{1},r_{2};k\right)\log\hat{f_{T}}\left(r_{1},r_{2};k\right).$$

The selection of intervals here, and thus the choice of *m*, is at the discretion of the user. It should be noted how this selection affects the outcome of the entropy calculation. In the extreme case, arbitrarily small interval sizes would allocate each observation to its own individual interval, with at most one occurrence in each interval. This results in a case of maximal entropy—the uniform distribution—which renders the exercise useless, since every portfolio would equally exhibit maximal entropy, $H(R_{P})=\log T$, for *T* unique states: one for each time period (T≤m). On the other extreme, arbitrarily large interval sizes would create one giant interval that encompasses every single observation. This results in a case of minimal entropy, $H(R_{P})=0$, with no randomness at all. This case is equally ineffective, as every portfolio would equally exhibit zero entropy. The user should explore reasonably sized intervals that yield the intended level of risk mitigation across portfolios. For more information on density estimation, please see Silverman (1998) [59], the spacing estimates method by Beirlant (1997) [60], and Learned–Miller (2003) [61] or the kernel density estimation method credited to Rosenblatt (1956) [62] and Parzen (1962) [63].

The above method can be extended to the case that there is a portfolio with *n* assets $R_{1},\ldots ,R_{n}$, and their actual returns over *T* time units, $r_{i}=\left(r_{i1},\ldots ,r_{iT}\right)$, i=1,…,n, with portfolio weights $w_{1},\ldots ,w_{n}$ such that $w_{1}+\cdots +w_{n}=1$.

It should also be noted that this empirical estimator of entropy is biased. In fact, it has been shown by Paninski (2003) [64] that there does not exist an unbiased estimator of entropy. Corrections to these estimators can be made, but they may not always be satisfactory, as shown by Miller (1955) [65]. In the case of this probability generating function, the severity of the bias depends on the interval selection and choice of *m*, with larger intervals leading to a stronger bias towards maximal entropy and smaller intervals leading to a stronger bias towards zero entropy, as described above.

### 3.4. Portfolio Entropy Objective Function

Extracting these coefficients, the exact portfolio entropy is given by the following portfolio entropy objective function:

(10) $$H\left(R_{P}\right)=-\frac{g^{\prime }\left(0\right)}{1!}\log\left(\frac{g^{\prime }\left(0\right)}{1!}\right)-\frac{g^{\prime \prime }\left(0\right)}{2!}\log\left(\frac{g^{\prime \prime }\left(0\right)}{2!}\right)-\cdots -\frac{g^{\left(m\right)}\left(0\right)}{m!}\log\left(\frac{g^{\left(m\right)}\left(0\right)}{m!}\right),$$

for *m* unique probability states, $A_{k}$. Each term in the objective function represents the respective term in the entropy function. This objective function is then to be minimized in the optimization problem.

The reason that this computation is so much easier than methods suggested by other authors is that the entropy is calculated directly on the end-state of the portfolio, *after* the allocation weights have been assigned. Other authors constructed the portfolio entropy by using convoluted combinations of the individual and conditional entropies—drastically increasing the complexity of the calculation as the number of assets *n* increases.

### 3.5. Return-Entropy Portfolio Optimization (REPO)

The new return-entropy portfolio optimization (REPO) problem uses a multi-objective function that minimizes entropy and maximizes expected returns, formulated as follows:

(11) $$\begin{array}{cc} \mathrm{minimize}\;\; & H\left(R_{P}\right)=-\sum_{k=1}^{m}\frac{g^{\left(k\right)}\left(0\right)}{k!}\log\left(\frac{g^{\left(k\right)}\left(0\right)}{k!}\right)\\ \mathrm{maximize}\;\; & E\left(R_{P}\right)=w_{1}E\left(R_{1}\right)+\cdots +w_{n}E\left(R_{n}\right)\\ \mathrm{subject}\;\;\mathrm{to}\;\; & w_{1}+\cdots +w_{n}=1,\;\;\;w_{i}\geq 0\;\;\forall \;i, \end{array}$$

for $R_{P}=w_{1}R_{1}+\cdots +w_{n}R_{n}$ and the *k*th-derivative at x=0 of the probability generating function:

(12) $$g\left(x;w_{1}r_{1}+\cdots +w_{n}r_{n}\right)=\frac{1}{T}\sum_{j=1}^{T}x^{\left\{k\;:\;a_{k-1}<\sum_{i=1}^{n}w_{i}r_{ij}\leq a_{k}\right\}}.$$

The reader should note that REPO evaluates the portfolio entropy as the *individual entropy of allocation-weighted portfolio returns* H(aX+bY) (a one-to-one dimensional function), whereas Philippatos [11] technically evaluated the portfolio entropy as the *joint entropy*H(aX,bY)=H(aX)+H(bY)−I(aX;bY) (an *n*-to-one dimensional function, for mutual information I(aX;bY)). However, the key point to note is that the probability generating function method used in REPO works perfectly fine for both H(aX,bY) and H(aX+bY).

Shown in the Appendix A, as a direct consequence of the well-known data processing inequality (Cover, 1991 [66], and Beaudry, 2012 [67]), H(aX,bY) is always greater than or equal to H(aX+bY)—which means more uniformity—due to the higher dimensionality of the former. To this end, it is our contention that more uniformity is worse for entropic portfolio optimization because with high enough dimensionality the distributions can quickly all resemble the uniform distribution (maximum entropy), and then no differentiation between portfolios via entropy can be done. Therefore, we decided to use H(aX+bY) as the portfolio entropy measure for the objective function in REPO.

## 4. A Portfolio Selection Example Using REPO

### 4.1. Data

In the example provided here, actual market data for ten randomly selected securities were gathered from the S&P/TSX 60 stock market index over the ten-year period from 1 January 2001, to 31 December 2010, totaling 520 data points each. Weekly closing prices were recorded and adjusted for stock splits, and relative weekly returns were computed as follows:

(13) $$r_{ij}=\frac{P_{ij}}{P_{i,j-1}}-1,$$

where $r_{ij}$ is the percent return on security *i* in period *j*, and $P_{ij}$ represents the price of security *i* in period *j*. For this example, the percent returns were discretized simply by using interval sizes of one basis point, with the minimum and maximum across all returns used as support boundaries; i.e., $\left[a_{min},a_{min}+1\right],\ldots ,\left[-2,-1\right],\left[-1,0\right],\left[0,1\right],\left[1,2\right],\ldots ,\left[a_{max}-1,a_{max}\right]$, all in units of basis points. According to this dataset, the minimum return across securities was −44 basis points and the maximum return was 42 basis points, for a total of m=86 possible probability states. The ten randomly selected securities and the sample means, variances, and entropies of their respective mean weekly returns over the ten-year period are presented below in Table 1, in which bps and nats are respective abbreviations of basis points and natural units.

The individual sample entropies are displayed here for demonstration purposes only—they are not actually used in REPO (as the portfolio entropy is calculated directly from the weighted portfolio data points). Notice how the trend validates the assumption that higher (absolute-value) return implies higher entropy: the sample correlation coefficient between absolute values of the sample means and the sample entropies in this sample is 0.880746. Interestingly, the rankings in terms of the sample variance or the sample entropy are almost identical here, except that Alimentation Couch-Tard Inc. and Manulife Financial Corp have swapped positions. The variance measure pegs Manulife as having significantly higher risk security (by almost 7 bps${}^{2}$), but according to the entropy Manulife has lower risk (by 0.05 nats).

### 4.2. Efficient Frontier and Portfolio Selection

In the portfolio selection problem, the efficient frontier refers to the set of the optimal portfolios that yield the greatest expected return for a defined level of risk, or alternatively the least risk for a defined level of expected return (the dual problem). The efficient frontier illustrates the risk–return trade-off for a given set of optimal portfolios.

**Figure 1:**
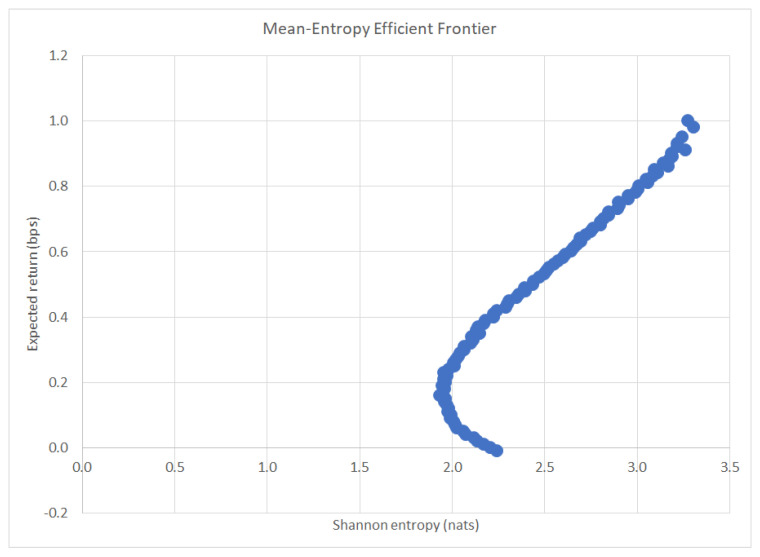
Mean-entropy efficient frontier.

The REPO algorithm was run on the data given in Section 4.1. Plotted below in Figure 1 is the mean-entropy efficient frontier among all possible optimal portfolio solutions. It is evident that greater risk—higher entropy—must be taken in order to achieve higher returns.

### 4.3. Comparison to MVPO

**Figure 2:**
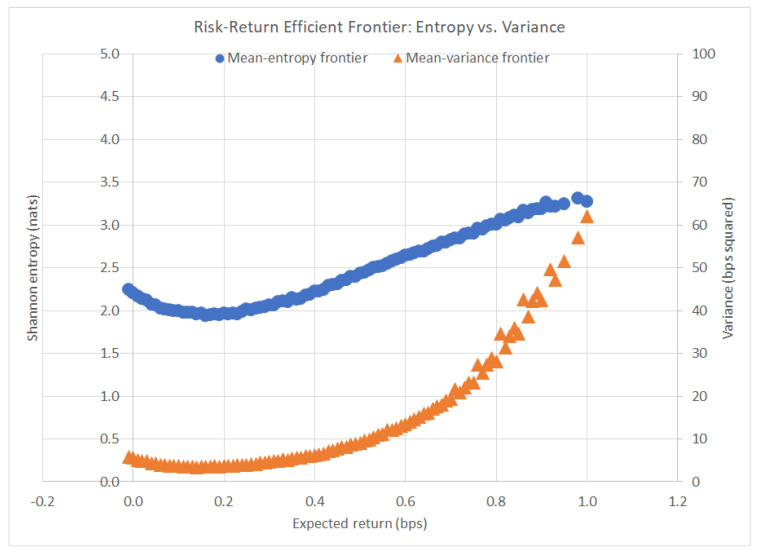
Risk–return efficient frontier: entropy vs. variance.

MVPO was run on the same data given in Section 4.1. Below, Figure 2 displays a translated plot of the mean-variance efficient frontier superimposed onto the mean-entropy efficient frontier. Notice the differences in the shape of the frontier: the variance curve is strictly convex, whereas the entropy curve is concave in the outer portion of the curve. This key difference is what enables REPO to find advantageous portfolio allocations that MVPO misses when it comes to balancing risk and reward.

**Figure 3:**
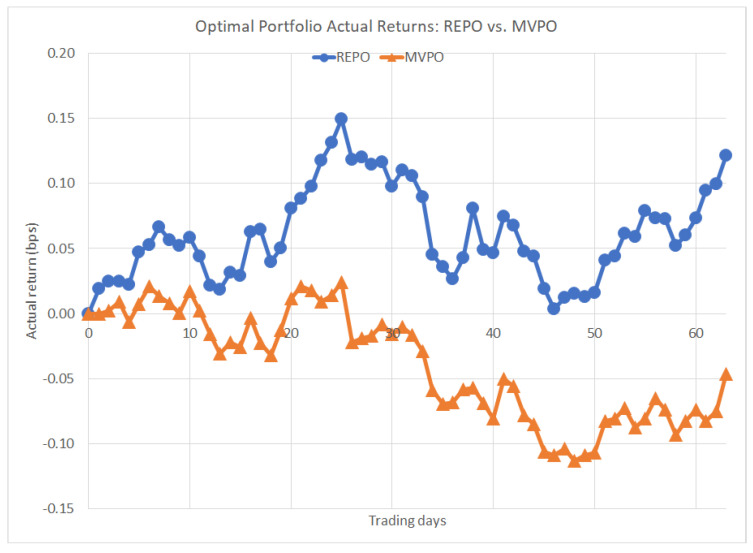
Optimal portfolio actual returns: return-entropy portfolio optimization (REPO) vs. mean-variance portfolio optimization (MVPO).

Another interesting observation is the fact that REPO and MVPO achieve their minimum risk portfolios at different optimal solutions and expected returns (Table 2):

Shown below are the differences in future, actual returns when using REPO versus using MVPO. Two portfolios were constructed using an expected return constraint equal to 0.37 bps and historical prices from 2001 to 2010: one minimum entropy portfolio using REPO and one minimum variance portfolio using MVPO. Optimal solutions to each strategy are shown in the following Table 3:

Forward-looking actual daily prices were then collected from the chosen ten securities starting 1 January 2011, and over the ensuing 13 weeks, actual portfolio performances were tracked and compared. The results are shown in Figure 3 below:

REPO outperformed MVPO here, with actual returns of 0.12 bps gain compared to −0.05 bps loss. Admittedly, this is only one case—which begs the question of how the methods would compare over the course of repeated trials. To that end, 7094 different portfolios were emulated, each with unique expected return constraints ranging from −0.0199 bps to 1.0036 bps. Two sets of portfolios were constructed in the same fashion as the previous example: one set of portfolios by minimizing the portfolio entropy using REPO, the other set by minimizing the portfolio variance using MVPO. The actual portfolio returns were calculated for each one of the 7094 portfolios and compared over the following 20 weeks. In 2925 of these emulations, both methods produced identical portfolios so that their returns were equivalent, but the remaining 4169 emulations each revealed a winning method. Table 4 below shows the results of the analysis, counting how many times each method won:

The emulation demonstrated that REPO outperformed MVPO handsomely in the near-to-medium term. After roughly four months, the REPO portfolios began to trail the MVPO portfolios, but in practice it is recommended that portfolios are balanced more frequently than once every four months. Therefore, REPO performs better than MVPO in the short-term time horizon.

### 4.4. Addressing the Five Main Issues with MVPO

This section explores how the REPO proposed solution handles the five main issues with MVPO mentioned in Section 2.

**Figure 4:**
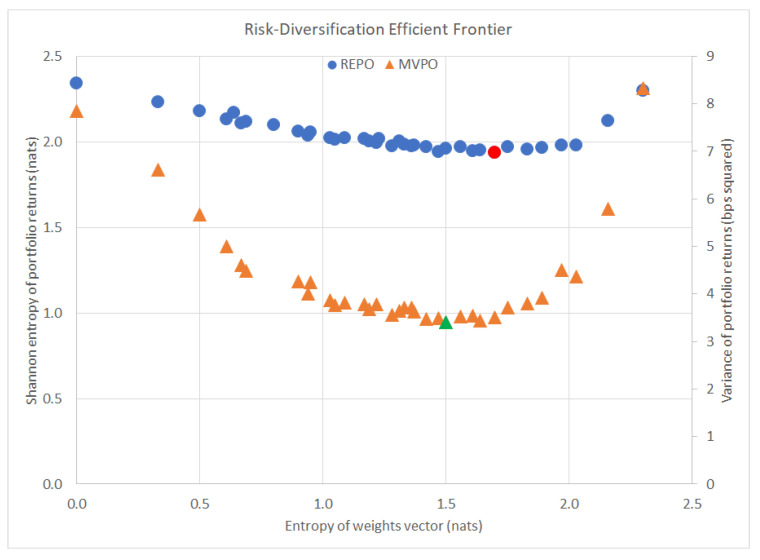
Risk-diversification efficient frontier.

(1) Large weights assigned to high risk assets. REPO lends itself very well to highly diversified portfolios, more-so than MVPO. Figure 4 below shows a plot of the most risk-averse portfolios according to each method and their corresponding diversification levels, measured by the Shannon entropy of the portfolio weight vector, H(x). The optimal minimum risk portfolio from MVPO is highlighted in green with a diversification level of only 1.5048 nats. The optimal minimum risk portfolio from REPO is highlighted in red with a diversification level of 1.6957 nats, almost 13% more diversified than the MVPO portfolio.

Additionally, the wider selection of lower risk portfolios from REPO tend to provide greater diversification levels than those of MVPO—as confirmed by the stronger inverse relationship: the correlation between REPO’s portfolio risk and diversification level is −0.5609, whereas the correlation between MVPO’s portfolio risk and corresponding diversification level is only −0.2772.

(2) Disturbing the dependence structure equilibrium. The dependence structure is well preserved when employing REPO in practice. REPO calculates the portfolio entropy directly in the objective function, as contributed by the securities and their respective weightings. This ensures the dependence structure equilibrium is maintained true to history (or assumptions). The dependence will be unaffected by any changes made to inputs. This is because the entropy of a random vector is not dependent on its mean in the same way that variance is. Consider two identical random vectors with equal mean, variance and entropy. Changing the value of a uniquely occurring element in one vector will change its mean and variance, but the entropy will be unchanged.

(3) Drastic variations in optimal solutions when adjusting inputs. Adjusting inputs to REPO yields expected and intuitive variations in optimal solutions. The multi-objective function of REPO can be formulated by combining the two objectives with a risk tolerance tuning parameter α:

(14) $$\mathrm{minimize}\;\;H\left(R_{P}\right)-\alpha E\left(R_{P}\right).$$

The sensitivity to changes in risk tolerance parameter α is low for the REPO method. The optimization was run on the same data using three different risk tolerance values: α = 1.0, 1.4, and 1.7. The optimal solutions were as follows Table 5:

(4) Dealing with returns that are non-normal or asymmetric. REPO handles non-normality and asymmetry with ease. There is no assumption or requirement for the data to be normally distributed or symmetric, and since there are no distribution assumptions at all, the optimization problem is fully non-parametric.

(5) Difficulty estimating covariance matrix and expected returns. REPO eliminates issues with estimating the covariance matrix and expected returns. It does all the risk calculations directly in the objective function, thus eliminating the need for any pre-calculations of covariance matrices or individual security variances. Referring back to (3), since the optimal solutions do not have drastic variations in adjustments to inputs, the pressure of making a sensible and accurate expected return estimate is reduced.

## 5. Conclusions

Presented here was a new entropy-based combinatorial approach to portfolio selection called return-entropy portfolio optimization (REPO) that addresses the five main practical concerns with MVPO: (i) optimal solutions assigning large allocation weights to high risk assets, (ii) disturbance of the assets’ dependence structure, (iii) drastic variations in optimal solutions when adjusting inputs, (iv) accommodating non-normal or asymmetric returns, and (v) difficulty estimating a covariance matrix and expected returns. By using combinatorial generating functions, REPO greatly simplifies the portfolio entropy computation. REPO is robust, non-parametric, and indifferent to non-normality and asymmetry, making it an ideal approach to the portfolio selection problem. In addition to these practical improvements over MVPO, REPO significantly outperforms the mean-variance method with greater future portfolio returns, especially in the short-term.

## 6. Materials and Methods

Data were sourced from Google Finance historical data extraction tool in Google Sheets for each equity. For example:

"=GOOGLEFINANCE("TSE:L","price","01/01/2001","12/31/2010","WEEKLY")".

Data and R code (R version 3.5.1) used for the portfolio selection example demonstrated in this paper can be accessed from the following DropBox sharing links: 

Data: https://www.dropbox.com/s/nd6lowuz5ngpjuf/SPTSX60.csv?dl=0

Code: https://www.dropbox.com/s/1v51xhako1jqkjh/SPTSX60-REPO.R?dl=0

## Figures and Tables

**Table 1:** The ten randomly selected securities from S&P/TSX 60 and the sample means, variances, and entropies of their mean weekly returns over the ten-year period.

Company Name	Ticker Symbol	Mean (bps)	Variance (bps${}^{2}$)	Entropy (nats)
Loblaw Companies Ltd.	L	0.006391	8.078711	2.381352
First Quantum Minerals Ltd.	FM	1.003592	61.97863	3.277249
Thomson Reuters Corp	TRI	−0.019931	11.65211	2.534170
Alimentation Couche-Tard Inc.	ATD.B	0.495919	17.89425	2.798943
Bank of Nova Scotia	BNS	0.242633	11.32819	2.466258
Teck Resources Ltd.	TECK.B	0.729174	60.76170	3.259236
Canadian Tire Corp Ltd.	CTC.A	0.284006	12.18994	2.605140
Inter Pipeline Ltd.	IPL	0.211462	7.847551	2.339923
Manulife Financial Corp	MFC	0.095557	24.68777	2.746475
Suncor Energy Inc.	SU	0.424803	27.36700	2.907254

**Table 2:** Minimum objective and optimal solutions for mean-variance portfolio optimization (MVPO) and return-entropy portfolio optimization (REPO) methods.

Method	Minimum Objective	Expected Return	Optimal Solution
MVPO	3.3993 bps${}^{2}$	0.1394 bps	(0.3,0.0,0.2,0.1,0.0,0.0,0.1,0.3,0.0,0.0)
REPO	1.9355 nats	0.1630 bps	(0.2,0.0,0.2,0.1,0.1,0.0,0.1,0.3,0.0,0.0)

**Table 3:** Optimal solutions for MVPO And REPO methods with expected returns of 0.37 bps.

Method	Expected Return	Optimal Solution
MVPO	0.37 bps	(0.0,0.1,0.0,0.4,0.0,0.4,0.0,0.0,0.1,0.0)
REPO	0.37 bps	(0.0,0.4,0.3,0.0,0.0,0.0,0.0,0.2,0.1,0.0)

**Table 4:** Comparison of REPO vs. MVPO portfolios over 20 weeks in 2011: number of portfolios that achieved greater returns.

	REPO	MVPO	Total	% REPO > MVPO
After 2 weeks	2377	1792	4169	57%
After 4 weeks	3115	1054	4169	75%
After 8 weeks	2537	1632	4169	61%
After 13 weeks	2345	1824	4169	56%
After 20 weeks	1699	2470	4169	41%

**Table 5:** Optimal solutions via REPO by various risk tolerances.

Risk Tolerance	Portfolio Entropy	Expected Return	Optimal Solution
α=1.0	1.9551 nats	0.2311 bps	(0.1,0.0,0.1,0.1,0.2,0.0,0.1,0.3,0.0,0.1)
α=1.4	2.1317 nats	0.3588 bps	(0.1,0.1,0.0,0.2,0.1,0.0,0.1,0.3,0.0,0.1)
α=1.7	2.1419 nats	0.3660 bps	(0.1,0.1,0.0,0.2,0.1,0.0,0.2,0.2,0.0,0.1)

## Abbreviations

The following abbreviations are used in this manuscript:

Bps	Basis points
CAPM	Capital asset pricing model
DEA	Data envelopment analysis
MED	Maximum entropy diversification
MQE	Mean-quadratic entropy
MSSS	Mean-semivariance-skewness-semikurtosis
MVPO	Mean-variance portfolio optimization
MVSE	Mean-variance-skewness-entropy
Nats	Natural units
PMPT	Post-modern portfolio theory
REPO	Return-entropy portfolio optimization

## Appendix A

Let the number of securities be *n* and the number of probability states be *m*. Then $\sum_{i=0}^{n-1}m^{i}$ individual and conditional entropies are required for computing the joint entropy.

**Proof.** The joint entropy of a portfolio with *n* securities can be expressed as a sum of their conditional entropies, defined as:

(A1) $$H\left(R_{1},\ldots ,R_{n}\right)=\sum_{i=1}^{n}H\left(R_{i}|R_{i-1},\ldots ,R_{1}\right)$$

We have the following claim: for any *n* securities and *m* probability states $A_{1},\ldots ,A_{m}$, with n,m∈N, $H(R_{1},\ldots ,R_{n})$ has $\sum_{i=0}^{n-1}m^{i}$ terms. We show it by induction on *n*. It is straightforward to show that the claim holds true for n=2 securities. Now we assume that the claim is true for *k* securities $H(R_{1},\ldots ,R_{k})$ that have $\sum_{i=0}^{k-1}m^{i}$ terms.

(A2) $$\begin{array}{cc} H(R_{1},\ldots ,R_{k},R_{k+1}) & =\sum_{i=1}^{k+1}H\left(R_{i}|R_{i-1},\ldots ,R_{1}\right)\\ & =\sum_{i=1}^{k}H\left(R_{i}|R_{i-1},\ldots ,R_{1}\right)+H\left(R_{k+1}|R_{k},\ldots ,R_{1}\right)\\ & =H\left(R_{1},\ldots ,R_{k}\right)+H\left(R_{k+1}|R_{k},\ldots ,R_{1}\right)\\ & =H\left(R_{1},\ldots ,R_{k}\right)+H\left(R_{k+1}|R_{k}=A_{1},\ldots ,R_{1}=A_{1}\right)+\cdots \\ & \;\;\;\;\;+H(R_{k+1}|R_{k}=A_{m},\ldots ,R_{1}=A_{m})\\ & \equiv \sum_{i=0}^{k-1}m^{i}\;\;\mathrm{terms}\;\;+\;\;m^{k}\;\;\mathrm{terms}\\ & =\sum_{i=0}^{k+1-1}m^{i}\;\;\mathrm{terms}. \end{array}$$

Thus, the claim holds true for any *n*. □

Let *X* and *Y* be two random variables that are not necessarily independent, and let *a* and *b* be scalar weights such that 0≤a,b∈R. Then, the joint entropy H(aX,bY) is always greater than or equal to the individual entropy H(aX+bY).

**Proof.** 

(A3) $$\begin{array}{cc} P(aX+bY=ax+by) & \geq P(aX=ax,bY=by)\\ \log P(ax+by) & \geq \log P(ax,by)\\ P(ax+by)\log P(ax+by) & \geq P(ax+by)\log P(ax,by)\\ P(ax+by)\log P(ax+by) & \geq P(ax,by)\log P(ax,by)\\ \sum_{i=1}^{n}\sum_{j=1}^{m}P\left(ax_{i}+by_{j}\right)\log P\left(ax_{i}+by_{j}\right) & \geq \sum_{i=1}^{n}\sum_{j=1}^{m}P\left(ax_{i},by_{j}\right)\log P\left(ax_{i},by_{j}\right)\\ -\sum_{i=1}^{n}\sum_{j=1}^{m}P\left(ax_{i}+by_{j}\right)\log P\left(ax_{i}+by_{j}\right) & \leq -\sum_{i=1}^{n}\sum_{j=1}^{m}P\left(ax_{i},by_{j}\right)\log P\left(ax_{i},by_{j}\right)\\ \Rightarrow H(aX+bY) & \leq H(aX,bY). \end{array}$$

This property can be easily extended to *n* variables. While it is true that the level of uniformity increases with less mutual information and decreases with greater mutual information, H(aX,bY) will certainly always have greater than or equal entropy to H(aX+bY). Note that this is basically a variation of the data processing inequality that states that no clever manipulation of the data can improve inference. See Cover (1991) [66] and Beaudry (2012) [67]. More formally, consider a probability model described by the Markov Chain: X→Y→Z, where X⊥Z|Y. Then it follows that I(X;Y)≥I(X;Z); i.e., no clever transformation of the received code *Y* can give more information about the sent code *X* than *Y* itself. In reference to the example provided, aX+bY is the so-called clever manipulation of the data, and it cannot ever exceed the information contained in the data pair (X,Y). □
